# Differential effects of psychological distress on mitigation and vaccination: A public health conundrum

**DOI:** 10.3389/fpsyg.2022.923056

**Published:** 2022-07-27

**Authors:** Joel Myerson, Michael J Strube, Leonard Green, Sandra Hale, Bridget Bernstein

**Affiliations:** Department of Psychological & Brain Sciences, Washington University in St. Louis, St. Louis, MO, United States

**Keywords:** COVID-19, public health, vaccination, mitigation, pandemic, distress

## Abstract

CDC-recommended mitigation behaviors and vaccination status were assessed in an online sample (*N* = 810; ages 18–80). Results were consistent with a differential distress hypothesis positing that whereas psychological distress, which is induced in part by social deprivation, interferes with mitigation behaviors involving social distancing, it motivates vaccination, in part because it, in turn, can increase social interaction. Age modulated these effects. Despite the greater risk of severe consequences, older adults not only showed less distress, but compared to younger participants with equivalent levels of distress, the older adults showed less effect of distress on both social distancing and vaccination status. Together these findings highlight a conundrum faced in public health messaging. Traditional “fear messages” may be less effective for older adults, who are most in danger, whereas in younger adults, the distress induced by fear messages may motivate vaccination but diminish mitigation behaviors needed to prevent subsequent “breakthrough” infections.

## Introduction

Americans overall have been more anxious and more depressed during the COVID-19 pandemic than previously [Center for Disease Control and Prevention (CDC), 2022]. More specifically, the increase in psychological distress associated with the pandemic appears to have been greater in younger adults than in older adults (Breslau et al., 2021), contrary to what one might have expected given the age-related differences in the personal consequences of COVID-19 infection (Williamson et al., 2020; Bosman et al., 2021; Lo et al., 2021). Appropriately enough, older adults also appear to be more likely to engage in CDC-recommended mitigation behaviors (e.g., social distancing) despite their lower levels of distress (Masters et al., 2020; Myerson et al., 2021). The possibility that infection could have more severe consequences for older adults does not appear to explain their mitigation behavior. Instead, people’s tendency to engage in such behavior appears to be independent not only of their concern regarding personal consequences of the pandemic, but also of their self-reported health, which is poorer in older adults, increasing their risk that an infection would have severe consequences (Myerson et al., 2021).

What does explain individual and age differences in mitigation behaviors? Perhaps surprisingly, people’s tendency to engage in social distancing appears to increase as their level of psychological distress decreases, and perhaps because distress appears to decrease with age, older adults are more likely to show social distancing than younger adults (Myerson et al., 2021). The relation between distress and mitigation could have important implications for public health messaging. That is, messages intended to increase anxiety and fear of the consequences of COVID-19 infection in order to motivate healthy, adaptive behavior could be counter-productive during the current pandemic because such messages may increase distress, thereby decreasing mitigation behaviors and thus actually increasing the risk of infection. For example, health anxiety, both general health anxiety and anxiety specific to COVID-19 infection, can induce hypervigilance, which while increasing detection of pandemic-related stimuli, might also lead to avoiding such stimuli, including public health messages, because they could further increase anxiety (Cannito et al., 2020).

But what about vaccination? Does the likelihood of getting vaccinated change with one’s level of distress, and is the mechanism underlying this relation the same as that underlying the relation between distress and mitigation behaviors? Although distress is associated with lower education levels and more distrust of vaccines (Ceccato et al., 2021), it is also associated with fear of infection. This could serve to motivate people to get vaccinated in order to potentially eliminate the possibility of infection as well as facilitate a return to pre-pandemic levels of social interaction. To address these issues, the present study focused first on the question of why there are age-related differences in psychological distress and mitigation behaviors (e.g., decreases in close proximity encounters). Is it because younger adults have been under more stress (Breslau et al., 2021), or is it because older adults are better at managing their stress (Carstensen et al., 2020; Burr et al., 2021)? And what is the source of this stress? Is it concern about the pandemic and its consequences, or is it an effect of social deprivation to which younger adults are more susceptible?

Our questions are inspired in part by our previous findings regarding the relations among age, distress, and mitigation (Myerson et al., 2021), but how robust are these associations? Do they replicate in a much larger sample, and do they generalize to other mitigation behaviors recommended by the CDC subsequent to our initial study, such as wearing masks and avoiding enclosed public spaces? In addition to the preceding questions, and perhaps most importantly, the present study also addresses the question of whether the findings with respect to mitigation generalize to individual and age differences in the decision to get vaccinated.

Previously, we hypothesized that social deprivation was a major source of psychological distress during the COVID-19 pandemic, and that such distress could interfere with mitigation behaviors because social distancing and other behaviors like mask-wearing might actually increase feelings of isolation and distress (Myerson et al., 2021). Importantly, this hypothesis further implies that distress would not interfere with vaccination because rather than increasing social deprivation, vaccination may decrease it. In fact, because of this, distress may actually have opposite effects on mitigation and vaccination. If true, this would make the task of public health messaging concerning COVID-19 even more challenging than it already is, as “breakthrough infections” suggest that vaccination does not eliminate the need for mitigation behaviors (Lo et al., 2021). In order to begin to address these issues and test what we term the *Differential Distress* hypothesis, the present study compared the effects of distress on mitigation behaviors and vaccination status in adults ranging in age from 18 to 80 years.

## Materials and methods

### Participants

Participants were recruited from April 16 to May 1, 2021, using the online platform Mechanical Turk (MTurk). (For a discussion of the strengths and weaknesses of this platform, see Goodman et al., 2012; see also Hauser and Schwarz, 2016). After indicating their consent online, which initiated the survey, 852 MTurk workers provided their responses and received $1.50 for their participation, which took 36.8 min on average. The submitted surveys were then screened for age, valid IP addresses associated with internet providers in the United States of America, and survey completion time so as to exclude those whose times were less than the time a fast, expert reader would require to read the survey questions (Rayner et al., 2016). Based on these criteria, data from 42 individuals were excluded from our analyses, six based on invalid US IP addresses and 36 based on their completion times, leaving 810 participants ranging in age from 18 to 80 years: 421 females and 383 males, plus six participants who did not report a gender (see Table 1). The racial and ethnic breakdown of these participants was 82.6% White, 8.3% Black, 4.9% Asian, and 4.2% other races; 14.6% identified as Hispanic/Latinx. The study was approved by the Institutional Review Board of Washington University in St. Louis.

**Table 1:** Age, number, gender, and income of participants.

Age range	*N*	Age: Mn (SD)	Percent female	Neighborhood income^a^
20–29.9	95	26.0 (2.59)	49.5	66,527
30–39.9	209	33.8 (2.59)	33.0	65,464
40–49.9	129	45.3 (3.08)	43.5	68,234
50–59.9	207	54.9 (2.82)	67.1	64,417
60–69.9	127	64.1 (2.97)	69.3	65,766
70+	42	71.9 (2.45)	52.3	61,802

a Income is based on the median incomes for participants’ zip codes as provided by the IRS.

### Procedure

The online survey consisted of three parts, beginning with questions about the frequency of four CDC-recommended mitigation behaviors and two mask-related behaviors. Specifically, the four mitigation behaviors included three Social Distancing behaviors and one Hygiene behavior: (1) being <6 feet from a person who was not a member of one’s household; (2) making physical contact with such a person; (3) being in an enclosed public space with other individuals; and (4) cleaning one’s hands with either sanitizer or soap and water. For each mitigation behavior, participants were asked about its frequency in three separate time frames, namely “on average this week,” “on average in November 2020 excluding Thanksgiving,” and “before the pandemic began.” The two mask-related behaviors were (1) being <6 feet from a person who was not a member of one’s household while wearing a mask and (2) being in an enclosed public space with other individuals while wearing a mask.

The second part of the survey consisted of all 14 items from the Hospital Anxiety and Depression Scale (HADS; Zigmond and Snaith, 1983), questions as to participants’ degree of concern about the possible effects of the pandemic on themselves and on others in their community, and questions regarding participants’ current levels of loneliness and social deprivation (Hughes et al., 2004). For example, the HADS asked participants questions like how often during the past week they “feel cheerful” and how often they feel “tense or wound up,” and the Loneliness scale asked questions like “how often do you feel left out” and “how often do you feel isolated from others.” Access to the complete survey may be obtained at osf.io/jmykd/.

Participants then were asked questions regarding personal connections with COVID-19 cases (e.g., number of acquaintances hospitalized with COVID-19 infections) and subjective opinions about vaccines against COVID-19 (e.g., “Do you believe vaccination against COVID-19 is safe?”).

The third part of the survey asked about religious identity and affiliation and the frequency of their attendance at religious gatherings, followed by a question concerning current vaccination status (“What is your current vaccination status: completely vaccinated, partially vaccinated, not yet vaccinated but likely to get vaccinated in the future, not likely to get vaccinated”). Participants then were asked to answer items from the IPIP-NEO (International Personality Item Pool-Neuroticism, Extraversion, Openness) personality test, followed by questions regarding their political affiliation and who they voted for in the 2020 United States Presidential Election. Finally, participants were asked about their gender, their age and date of birth (for verification), their ethnicity and race, and their home zip code.

## Results

### Mitigation

Our analyses of age and individual differences in mitigation behaviors concerned three questions. Do the present results replicate previous findings (Myerson et al., 2021)? Do these findings generalize to new measures of mitigation behavior that may also be somewhat socially isolating? And what do the present results tell us about the mechanisms underlying age and individual differences in mitigation behaviors?

#### Replication and generalization

As in our previous study of CDC-recommended mitigation behaviors (Myerson et al., 2021), social distancing, measured as decreases in the frequency of close proximity interactions (Prox) and physical contact (Cont) with non-household members, increased significantly with age, whereas hand hygiene (Hand; increases in handwashing and disinfecting) did not. Both anxiety and depression were negatively correlated with age and social distancing measures, further replicating our previous findings. Because anxiety and depression scores on the Hospital Anxiety and Depression Scale (HADS) were strongly correlated (*r* = 0.637), we used total HADS scores as a measure of psychological distress in our analyses; similarly, we used the total score for mask-wearing (Msks) because of the high correlation (0.597) between the two mask-wearing measures.

Since our previous study was published, the CDC recommended three new mitigation measures (i.e., minimizing visits to enclosed public places (Publ) and also mask wearing during close-proximity interactions and in enclosed public spaces) that we hypothesize might be somewhat socially isolating, and thus related to distancing. These new mitigation measures also increased with age and decreased with psychological distress, and were significantly correlated with the other distancing measures, suggesting that the pattern of interrelations among age, distress, and social distancing represents a general phenomenon (see Table 2).

**Table 2:** Intercorrelations (with probabilities in italics) of mitigation behaviors, psychological distress (HADS), and age.

Variable	Prox	Cont	Hand	HADS	Age	Publ	Msks
Prox	—						
	—						
Cont	0.399	—					
	*<0.001*	*—*					
Hand	0.093	0.129	—				
	*0.011*	*<0.001*	*—*				
HADS	−0.261	−0.241	−0.072	—			
	*<0.001*	*<0.001*	*0.045*	*—*			
Age	0.205	0.133	0.018	−0.269	—		
	*<0.001*	*<0.001*	*0.627*	*<0.001*	*—*		
Publ	0.394	0.375	0.121	−0.198	0.199	—	
	*<0.001*	*<0.001*	*<0.001*	*<0.001*	*<0.001*	*—*	
Msks	0.105	0.131	0.210	−0.091	0.178	0.066	—
	*0.001*	*<0.001*	*<0.001*	*0.008*	*<0.001*	*0.035*	*—*

Prox is the frequency with which one was <6 feet from a person who was not a member of one’s household; Cont is the frequency with which one made physical contact with such a person; Hand is the frequency with which one cleaned one’s hands with either sanitizer or soap and water; Publ is the frequency with which one was in an enclosed public space with other individuals; Msks is the frequency with which one wore a mask in an enclosed public space. All self-reported frequencies referred to the past week.

#### Mechanisms

The intercorrelations among mitigation behaviors, age, and HADS scores raise the question of whether younger adults are less likely to engage in social distancing simply because they are more distressed. That is, does the negative correlation between age and distress explain the negative correlation between age and distancing, or do age and distress play independent roles?

In order to examine the contributions of HADS and Age to both mitigation behaviors and vaccination using the same analytic approach, logistic regression analyses were conducted, revealing first that HADS and Age made independent contributions to the prediction of the three social distancing measures: decreased frequency of close proximity interactions and physical contact with non-household members and visits to enclosed public spaces (see Table 3). Note that the coefficient for HADS was negative in each case, indicating that even with the effects of age controlled, greater distress was associated with a decreased likelihood of social distancing. When a similar analysis was conducted with Vaccinated, either partially or fully, as a binary dependent variable, the coefficients for age and HADS were again significant, but unlike with social distancing, the HADS coefficient was positive, indicating that the likelihood of vaccination increased with distress, consistent with the Differential Distress hypothesis.

**Table 3:** Logistic regression analyses of the contributions of age and distress to social distancing and vaccination.

**Dependent: Proximity**	***χ***^**2**^ **= 70.17, *p* < 0.001**				
	***b***	**SE**_***b***_	**Std. Coeff.**	***Z***	***p***
Intercept	0.50	0.35	0.79	1.43	0.153
Age	0.02	0.01	0.35	4.07	<0.001
HADS	−0.06	0.01	−0.52	5.93	<0.001
**Dependent: Contact**	***χ***^**2**^ **= 50.13, *p* < 0.001**				
	***b***	**SE**_***b***_	**Std. Coeff.**	***Z***	***p***
Intercept	0.89	0.34	0.62	2.62	0.009
Age	0.01	0.01	0.16	2.03	0.043
HADS	−0.06	0.01	−0.49	−5.84	<0.001
**Dependent: Public Space**	***χ***^**2**^ **= 50.19, *p* < 0.001**				
	***b***	**SE**_***b***_	**Std. Coeff.**	***Z***	***p***
Intercept	0.03	0.33	0.61	0.08	0.938
Age	0.03	0.02	0.35	4.32	<0.001
HADS	−0.04	0.01	−0.34	−4.24	<0.001
**Dependent: Vaccinated**	***χ***^**2**^ **= 23.49, *p* < 0.001**				
	***b***	**SE**_***b***_	**Std. Coeff.**	***Z***	***p***
Intercept	−0.48	0.33	0.78	−1.44	0.150
Age	0.01	0.01	0.19	2.34	0.019
HADS	0.05	0.01	0.38	4.65	<0.001

Std. Coeff. refers to the standardized coefficient.

### Vaccination

Our analyses of age and individual differences in vaccination decisions began by considering whether or not mitigation and vaccination decisions involved similar mechanisms. This was done first by comparing vaccinated and unvaccinated participants and then in more detail by comparing participants of different vaccination status: the fully vaccinated, the partially vaccinated, unvaccinated participants who said they were likely to get vaccinated in the future, and unvaccinated participants who said future vaccination was unlikely. Our final set of analyses focused directly on the Differential Distress hypothesis, first by examining the role of social deprivation in psychological distress and then by examining the extent to which vaccination decreased social deprivation.

#### Vaccinated or not?

Vaccinated and unvaccinated participants differed significantly in their level of distress and (not surprisingly) in their attitudes toward vaccination, as well as in a number of other measures. Examination of the intercorrelations among these measures, however, revealed potential sources of multicollinearity, which lead to the formulation of a reduced logistic regression model (see Table 4; a complete correlation matrix, along with a table with descriptive measures, and raw data are available at osf.io/jmykd/; differential effects of psychological distress on mitigation and vaccination: A public health conundrum). Along with other measures showing significant differences between vaccinated and unvaccinated, this model included HADS rather than its strong correlates (conscientiousness and emotional stability) because of the importance of identifying simple constructs potentially manipulable by public health efforts (e.g., selection of state over trait measures) and also because of the fundamental role played by psychological distress in our theorizing.

**Table 4:** Correlations (with probabilities in italics) among the measures of the reduced model of vaccination.

Hosp	Vacc	Relig	HADS	Age	
Hosp	—				
	—				
Vacc	−0.118	—			
	*0.002*	*—*			
Relig	0.450	−0.295	—		
	*<0.001*	*<0.001*	*—*		
HADS	0.239	−0.335	0.238	—	
	*<0.001*	*<0.001*	*<0.001*	*—*	
Age	−0.126	0.258	−0.233	−0.268	—
	*<0.001*	*<0.001*	*<0.001*	*<0.001*	*—*

Hosp is the number of acquaintances hospitalized with COVID-19; Vacc represents attitudes regarding vaccination; Relig is the number of religious services attended per month.

Both age and HADS were still positively associated with vaccination after controlling for other differences between vaccinated and unvaccinated participants (see Table 5). Nevertheless, the data for the subset of 734 participants who voted for either Trump or Biden in the 2020 presidential election were also examined because of previous reports of political differences in vaccination (e.g., Bolsen and Palm, 2021). Consistent with these reports, 53.5% of Trump voters in the present sample were vaccinated, whereas 78.7% of Biden voters were vaccinated. Biden voters had significantly more positive attitudes toward vaccination, estimated that they and their acquaintances had a greater chance of infection, and were more distressed and had greater concerns about the consequences of the pandemic. Perhaps unexpectedly, Biden voters in our sample attended religious services more often than Trump voters, and Trump voters had significantly higher scores on the Conscientiousness and Emotional Stability scales of the IPIP NEO test. The only measure on which they did not differ significantly was the number of acquaintances who had been hospitalized because of COVID-19.

**Table 5:** Logistic regression analysis predicting vaccination.

**Model**	**df**		***χ*** ^**2**^	***p***	
H₀	665				
H₁	659		248.62	<0.001	
	***b***	**SE**_***b***_	**Std. Coeff.**	***Z***	***p***
Intercept	−7.57	0.77	0.99	−9.83	<0.001
Hosp	0.31	0.07	1.73	4.53	<0.001
Vacc	0.27	0.03	1.38	10.18	<0.001
Relig	0.13	0.04	0.99	3.46	<0.001
Chance 0.21	0.07	0.36	2.99	0.003	
HADS	0.05	0.01	0.30	2.57	0.010
Age	0.03	0.01	0.36	3.38	<0.001

Hosp is the number of acquaintances hospitalized with COVID-19; Vacc represents attitudes regarding vaccination; Relig is the number of religious services attended per month; Chance is estimated likelihood of infection.

Despite the multiple differences between Biden and Trump voters, a logistic regression analysis examining the roles of Age, Distress, and political preference revealed that although all three variables were significant predictors of vaccination, of the possible two-way and three-way interactions, only the interaction of Age and Distress was significant (see Table 6), suggesting that despite the differences between Trump and Biden voters, the mechanisms underlying vaccination were similar for both. The interaction of Age and Distress reflects the fact that as Age increased, the rate at which the probability of vaccination increased with the level of distress (HADS) decreased, a finding examined in detail below in light of the additional insights provided by multinomial regression.

**Table 6:** Logistic regression analysis of vaccinated vs. unvaccinated with age, distress, and political preference as independent variables.

	*b*	SE*_b_*	*Z*	*p*
Intercept	1.35	0.11	12.17	<0.001
HADS	0.04	0.01	3.86	<0.001
Age	0.02	0.00	2.69	0.007
2020Vote	−1.19	0.18	−6.64	<0.001
HADS × Age	−0.00	0.00	−3.95	<0.001
HADS × 2020Vote	−0.02	0.02	−0.98	0.326
Age × 2020Vote	−0.02	0.01	−1.47	0.140
HADS × Age × 2020Vote	−0.00	0.00	−1.02	0.307

2020Vote is a binary variable distinguishing between Trump and Biden voters (Biden = 0, Trump = 1). Table values represent stage that a predictor was tested in a hierarchical regression model: main effects, then addition of two-way interactions, then the addition of the three-way interaction. This ensures that predictors are controlled for other predictors of equal or lesser complexity.

Political preferences, of course, are not the only basis for subdividing the present sample. Another important way to subdivide the sample for public health purposes is based on vaccination status, distinguishing not only between fully and partly vaccinated, but also between unvaccinated participants who reported they were likely to get vaccinated later and those who reported they were unlikely to do so. Multinomial regression, a generalization of logistic regression to multiclass problems, was used to identify measures that distinguished reference groups (fully vaccinated participants in the first analysis and participants who said they were unlikely to get vaccinated in the second analysis) from those who differed in vaccination status (see Table 7). The same reduced model, but with a HADS × Age interaction term added based on the preceding analysis, was used to minimize multicollinearity.

**Table 7:** Multinomial analysis of groups differing in vaccination status with fully vaccinated and unlikely to get vaccinated as reference (REF) categories.

REF: Fully	*b*	SE*_b_*	*Z*	*p*	REF: Unlikely	*b*	SE*_b_*	*Z*	*p*
Unlikely					Unlikely				
Intercept	−3.06	0.35	−8.70	<0.001	Intercept	**—**	**—**	**—**	**—**
Hosp	−0.41	0.13	−3.05	0.002	Hosp	**—**	**—**	**—**	**—**
Vacc	−0.62	0.07	−9.02	<0.001	Vacc	**—**	**—**	**—**	**—**
Relig	−0.08	0.06	−1.46	0.144	Relig	**—**	**—**	**—**	**—**
Chance	−0.48	0.14	−3.50	<0.001	Chance	**—**	**—**	**—**	**—**
HADS	−0.06	0.03	−2.36	0.018	HADS	**—**	**—**	**—**	**—**
Age	−0.02	0.01	−1.30	0.193	Age	**—**	**—**	**—**	**—**
HADS × Age	0.00	0.00	1.64	0.102	HADS × Age	**—**	**—**	**—**	**—**
Likely					Likely				
Intercept	−1.02	0.16	−6.17	<0.001	Intercept	2.04	0.37	5.52	<0.001
Hosp	−0.31	0.08	−3.72	<0.001	Hosp	0.10	0.15	0.68	0.499
Vacc	−0.16	0.04	−4.51	<0.001	Vacc	0.46	0.07	7.01	<0.001
Relig	−0.12	0.05	−2.69	0.007	Relig	−0.04	0.07–0.64	0.520	
Chance	−0.10	0.09	−1.16	0.245	Chance	0.38	0.14	2.79	0.005
HADS	−0.03	0.02	−1.85	0.064	HADS	0.03	0.03	1.15	0.249
Age	−0.04	0.01	−4.40	<0.001	Age	−0.02	0.01	–1.52	0.129
HADS × Age	0.00	0.00	2.46	0.014	HADS × Age	0.00	0.00	–0.05	0.957
Partly					Partly				
Intercept	−0.52	0.12	−4.42	< 0.001	Intercept	2.54	0.36	7.12	<0.001
Hosp	−0.02	0.02	−0.77	0.442	Hosp	0.39	0.13	2.94	0.003
Vacc	0.02	0.03	0.78	0.435	Vacc	0.64	0.07	9.11	<0.001
Relig	0.01	0.01	0.53	0.599	Relig	0.09	0.06	1.57	0.117
Chance	−0.10	0.08	−1.32	0.188	Chance	0.38	0.14	2.69	0.007
HADS	0.01	0.02	0.69	0.493	HADS	0.08	0.03	2.66	0.008
Age	−0.02	0.01	−2.83	0.005	Age	−0.00	0.02	−0.30	0.768
HADS × Age	0.00	0.00	0.72	0.470	HADS × Age	−0.00	0.00	−1.19	0.233
Fully					Fully				
Intercept	**—**	**—**	**—**	**—**	Intercept	3.06	0.35	8.70	<0.001
Hosp	**—**	**—**	**—**	**—**	Hosp	0.41	0.13	3.05	0.002
Vacc	**—**	**—**	**—**	**—**	Vacc	0.62	0.07	9.02	<0.001
Relig	**—**	**—**	**—**	**—**	Relig	0.08	0.06	1.46	0.144
Chance	**—**	**—**	**—**	**—**	Chance	0.48	0.14	3.50	<0.001
HADS	**—**	**—**	**—**	**—**	HADS	0.06	0.03	2.36	0.018
Age	**—**	**—**	**—**	**—**	Age	0.02	0.01	1.30	0.193
HADS × Age	**—**	**—**	**—**	**—**	HADS × Age	−0.00	0.00	−1.64	0.102

Hosp is the number of acquaintances hospitalized with COVID-19; Vacc represents attitudes regarding vaccination; Relig is the number of religious services attended per month; Chance is estimated likelihood of infection.

The four vaccination status groups differed in many respects, as indicated by the many independent variables with significant coefficients. The most important differences from a public health perspective, however, are probably those that may be modifiable by messaging or other interventions so as to move individuals in the two unvaccinated categories into the Fully vaccinated category or to move those in the Unlikely to get vaccinated category into the Likely to get vaccinated group.

Two things distinguished participants in both of the unvaccinated status categories from the Fully vaccinated. First, number of acquaintances hospitalized (Hosp) because of COVID-19, which was lower than for the Fully Vaccinated; second, unsurprisingly, attitude toward vaccination (Vacc) was more negative than for the Fully vaccinated, and especially so for the Unlikely group. While both unvaccinated groups reported less distress than the Fully vaccinated, the HADS coefficient was significant for the Unlikely group, whereas with the Likely group, it was the HADS × Age interaction. Relative to an Unlikely reference, the Likely also differed in that their estimates of the chance of infection (Chance) were not as low and their Vacc score was not as negative.

**Figure 1:**
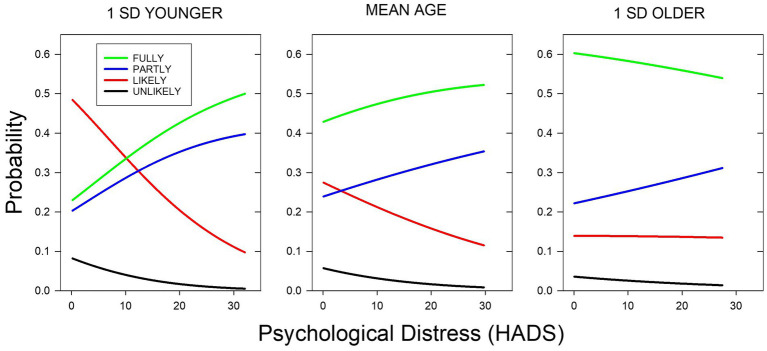
Probability of vaccination status as a function of HADS score for a participant whose age was the sample mean (46.9  years, center panel) and for participants 1 SD younger and older than the mean (ages 32.8 and 61.0  years, left and right panels, respectively). The probabilities of a person’s status being Unlikely, Likely, Partially, or Fully are represented by black, red, blue, and green lines, respectively. Vaccination status probabilities are depicted for HADS scores up to 2 SDs above the mean for each of the ages represented.

Based on the multinomial analyses, it is possible to visualize the intersectionality of age, distress, and vaccination status. Figure 1 depicts the probability of each vaccination status for a participant whose age was one standard deviation below the mean age for the sample (left panel), a participant who was of average age (center panel), and one whose age was one standard deviation greater than the sample mean (right panel). In the first two cases, as may be seen, the probability of full or partial vaccination increased with Distress, whereas the probability of not being vaccinated decreased, and both the increases and decreases were steeper for the average younger participant. In contrast, the probabilities changed relatively little with the level of distress for an older participant, although it should also be noted that, compared with a younger participant, an older participant’s probability of being fully vaccinated was greater and their level of distress was lower.

#### Differential effects of distress

Further analyses were conducted to test the two components of our hypothesis regarding the differential effects of distress on mitigation and vaccination. That is, we hypothesized that social deprivation was a major cause of psychological distress during the COVID-19 pandemic. We hypothesized further that if social distancing increased feelings of isolation, it could lead to a reluctance to engage in distancing in people who already were distressed, whereas because vaccination offered the promise of a return to previous levels of socialization, distress might actually increase the likelihood of vaccination. Indeed, distress levels (HADS scores) were negatively correlated with distancing but positively correlated with vaccination, although the strength of this relation decreased with age.

Consistent with our hypothesis regarding the social source of psychological distress during the COVID-19 pandemic, distress (HADS) was significantly correlated with loneliness (Lone; *r* = 0.528, *p* < 0.001). Multiple regression analyses were used to assess the relative contributions of loneliness and the other pandemic-related concerns identified in our previous comparison of vaccinated and unvaccinated participants: concerns about the consequences of the pandemic (Concerns), both personal and community-related; estimates of the likelihood of infection (Chance); and the number of acquaintances hospitalized (Hosp). This model accounted for 44.0% of the variance in HADS scores (see Table 8). A reduced model with only the two significant predictors, Lone and Chance, accounted for 43.4% of the variance: Lone and Chance uniquely accounted for 18.9% and 15.5% of the variance, respectively, and 9.0% was shared.

**Table 8:** Multiple regression analysis of the contributions of pandemic-associated concerns to psychological distress (HADS).

	*b*	SE*_b_*	Std. Coeff.	*t*	*p*
Intercept	−4.42	0.93		−4.78	<0.001
Lone	2.18	0.13	0.48	16.52	<0.001
Chance	1.58	0.18	0.33	8.88	<0.001
Hosp	0.08	0.04	0.06	1.94	0.053
Concerns	0.03	0.05	0.03	0.72	0.470

Lone is score on the Loneliness scale; Chance is estimated likelihood of infection; Hosp is number of acquaintances hospitalized with COVID-19; Concerns represents personal- and community-related regarding the consequences of the pandemic.

These results are consistent with the first component of our differential distress hypothesis in which social deprivation leads to distress, which may interfere with mitigation behaviors like social distancing that can exacerbate feelings of isolation. The second component of our hypothesis, of course, is that more distressed people, who as a result are less likely to engage in distancing, are more likely to be vaccinated. And indeed, further analyses revealed that vaccination does lead to an increase in social behavior. In addition to being asked about their mitigation behaviors when the survey was administered in April 2021, participants were also asked to recall their mitigation behaviors in November of 2020, before vaccines were available. Thus, for those who reported being vaccinated at the time of the survey, the frequencies of close encounters could be compared before and after vaccination, making it possible to examine the role of vaccination as a predictor of mitigation behavior, reversing the relation examined previously. Moreover, the behavior of the fully vaccinated could be compared with that of the partially vaccinated for whom social interactions were not yet as safe.

**Figure 2:**
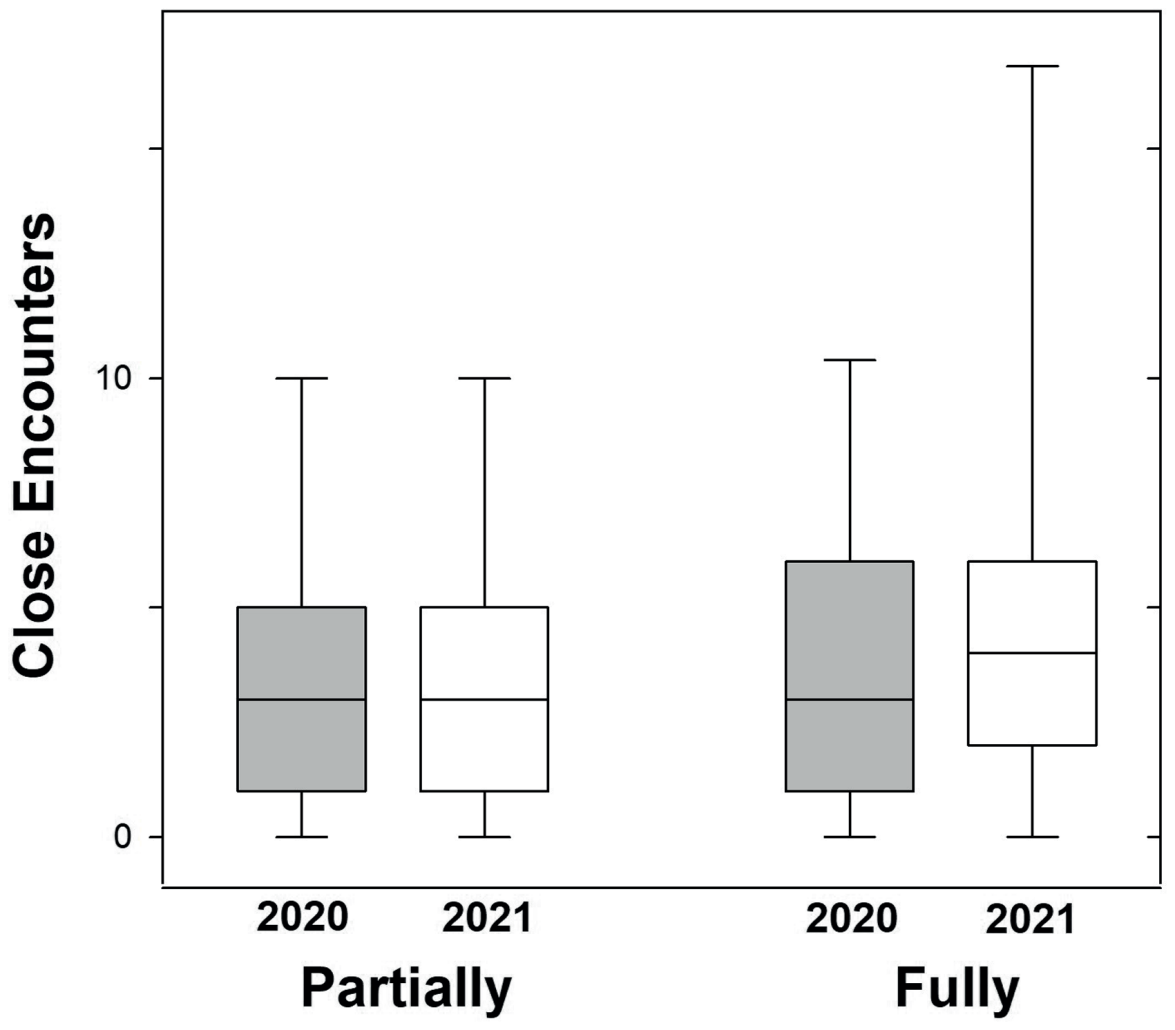
Box plots of the frequency of close proximity interactions (number per week) by partially and fully vaccinated participants. In 2020, neither group had been vaccinated; in 2021, the partially vaccinated participants were not yet protected from COVID-19 infection, whereas the fully vaccinated participants were protected.

The results of this comparison (Figure 2) are consistent with the hypothesis that whereas distress interferes with social distancing, it motivates vaccination by permitting social interactions. Signed Rank tests revealed that fully vaccinated participants increased their close proximity interactions following vaccination (*W* = 7306.00, *p* = 0.001), but the partially vaccinated did not (*W* = 910.00, *p* = 0.314), so that the proportion of fully vaccinated participants who had fewer close encounters at the time of the survey than before the pandemic (0.598) was lower than the proportion of partially vaccinated participants (0.698).

## Discussion

It is well established that social deprivation can lead to psychological distress as evidenced by symptoms of depression and anxiety like those assessed here (Beutel et al., 2017; Cavicchioli et al., 2021). Psychological distress can have other causes, of course, and the COVID-19 pandemic is certainly associated with numerous other sources of distress. Accordingly, one goal of the present study was to compare the relative contributions of loneliness to various other pandemic-related sources of distress. Another goal was to examine not just the causes of distress, but its consequences as well.

In a previous study conducted prior to the development of effective vaccines against the COVID-19 (Myerson et al., 2021), we observed that psychological distress appeared to interfere with CDC-recommended mitigation behaviors, particularly those that could be thought of as “socially-distancing.” We hypothesized that such behaviors could exacerbate the feelings of social isolation that people might be experiencing, and the more distressed they were, the more likely they were to avoid making things worse. Therefore, we suggested that distress-inducing “fear messages” highlighting potentially serious consequences of COVID-19 might be contraindicated if the aim was to promote mitigation.

An obvious implication of this hypothesis is that, in contrast to their negative effects on mitigation behaviors, fear messages and psychological distress might actually increase the likelihood of vaccination because it offers a return to more normal levels of social interaction. The idea that social deprivation leads to distress, which has contrasting effects on mitigation and vaccination because they can have opposite effects on social deprivation, constitutes the Differential Distress hypothesis.

**Figure 3:**
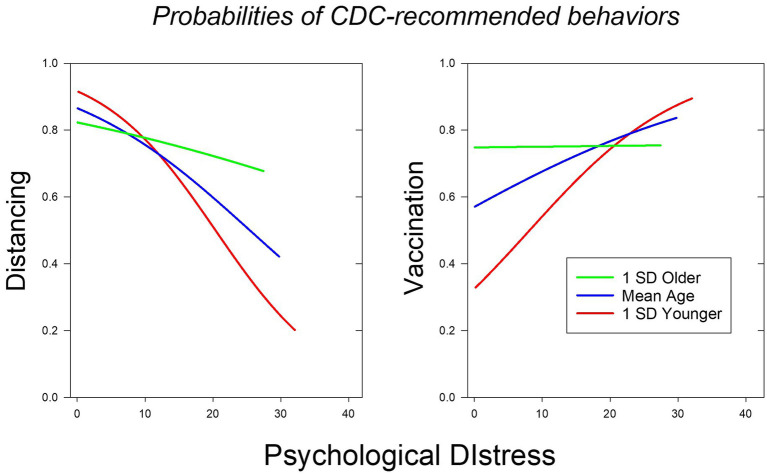
The probability that a participant decreased the frequency of close interactions (left panel) and the probability that they were vaccinated (right panel) as a function of their age and HADS score. The blue, red, and green lines depict the probabilities for a participant of average age in the sample, one whose age was 1 SD below the mean, and one whose age was 1 SD above the mean, respectively. For each age, probabilities are depicted for HADS scores up to two SDs above the mean (maximum possible score = 42).

The present findings with respect to mitigation both prior to and during the pandemic are consistent with this hypothesis (Figure 3). We began by replicating the finding that individual and age differences in distress predicted the degree to which participants followed CDC recommendations with respect to social distancing, and then showed that this finding generalized to their new recommendations regarding visits to enclosed public spaces and, to a lesser extent, to mask-wearing. Moreover, the contributions of age and psychological distress to social distancing behaviors proved to be both independent and opposite in sign. These results indicate that the increases in mitigation with age were not simply due to the age-related decrease in distress levels, which could reflect a general ability of older adults to cope with daily stressors as revealed in pre-pandemic studies (Carstensen et al., 2020; Burr et al., 2021). However, the age-related increases in mitigation do not appear to simply reflect increases in compliance, given that hand-hygiene, another mitigation behavior strongly recommended by the CDC, did not covary with age.

The results for social distancing behaviors stand in stark contrast to the results for vaccination (Figure 3). Although age and psychological distress made independent contributions to vaccination and the likelihood of both mitigation and vaccination increased with age, greater distress decreased the likelihood of social distancing, whereas it increased the likelihood of vaccination. This finding is consistent with our Differential Distress hypothesis, but it creates a conundrum from a public health perspective. This conundrum would not exist if vaccinations were permanently effective but given the prevalence of breakthrough infections in the vaccinated, as well as the present and possibly future emergence of vaccine-resistant COVID-19 variants, vaccination cannot be assumed to preclude the need for social distancing.

“Fear messages” warning of the dangers of non-compliance with public health recommendations are known to be more effective when the recommended behaviors are one-time events, as vaccinations often are. They function by both creating distress and indicating a way to relieve that distress. In the present case, however, the opposite effects of psychological distress suggest that messages that raise distress levels run the risk of increasing vaccination likelihood while at the same time decreasing the likelihood of social distancing, a trade-off that while it may have seemed desirable previously, currently seems less so.

Multinomial analyses focused on differences between groups who differed in their vaccination status provided a more nuanced view of the present data, suggesting targets for public health efforts. For example, unvaccinated participants who said they were likely to get vaccinated in the future differed from those who said they were unlikely both in their estimates of the chance of infection with COVID-19 and in their scores on the Vacc scale, which measured attitudes toward vaccination. When responses to individual items on the Vacc scale were examined to define potential targets more clearly, it turned out that although there were no group differences with respect to the (in)convenience of vaccination, both unvaccinated groups rated vaccination safety and the likelihood of infecting others lower than the vaccinated groups, although these differences were larger for the Unlikely group. Thus, efforts to change attitudes regarding vaccination and to increase estimates of the infectiousness of the COVID-19 virus may be a productive route for moving individuals from the Unlikely group to the Likely group. Notably, the Unlikely group indicated “personal, cultural, or religious objections to vaccination against COVID-19” to a greater degree than the other three groups, although because they were not asked to specify these objections, more information undoubtedly will be needed in order to target them effectively.

While the opposite effects of distress on mitigation and vaccination provide the name and the primary empirical basis for the Differential Distress hypothesis, the idea that much of the distress during the current pandemic is caused by social deprivation provides the conceptual basis. Not only has it long been known that social deprivation, as exemplified by quarantines during previous pandemics, can lead to psychological distress as evidenced by symptoms of depression and anxiety (for a review, Henssler et al., 2021), but perhaps not unexpectedly, that effect has been exacerbated by the COVID-19 pandemic with the resultant increases in health anxiety (Kämpfen et al., 2020; Okruszek et al., 2020; Palgi et al., 2020; Tyrer, 2020).

Our full idea, of course, is that some people choose not to engage in mitigation behaviors that might increase their distress by constraining their interactions with others (social distancing), whereas they will choose other behaviors that can increase their interactions (getting vaccinated) and thereby decrease their distress. The present study provides empirical support for both parts of this idea, first in the finding that distress is not mostly due to pandemic concerns but instead that loneliness plays a major role, and second in the finding that once vaccinated, people tend to increase their close proximity interactions with others from outside their household.

The present study is, of course, not without limitations. For example, it relies on self-reported frequencies of past behavior (e.g., during the previous November and before the pandemic). Additional evidence would be provided by a study that assessed distress both before and after vaccination, rather than just after, as in the current study. Although the present results replicated (and extended) previous findings on mitigation, both samples were drawn from the same source (MTurk) and replication of the present results with participants from different sources could help establish the robustness of these findings. Much larger samples, moreover, might provide the power needed to examine similarities and differences among demographic groups.

Recent history, of course, suggests that the COVID-19 pandemic itself, rather than methodological and theoretical concerns, may tell us what specific studies will need to be done next. The present results, however, shed light on the nature of the public health problem to be faced and provide a perspective that may be generally useful. Taken together, the present findings reveal that the current conundrum has two components. First, psychological distress is a two-edged sword, interfering with some behaviors and facilitating others, and the trade-offs need to be kept in mind during the current pandemic. Second, the dual effects of distress are manifested most strongly in younger adults, and older adults may not be as affected by messages intended to affect distress levels to motivate health behavior.

With respect to the first component, one potential approach would be to emphasize prosocial appeals rather than merely appealing to self-interest because the former may induce less distress than the latter, which highlights the dire consequences of unhealthy behavior. Research suggests that both mitigation and vaccination may be motivated by their benefits to others (Ebrahimi et al., 2021; Jordan et al., 2021; Böhm and Betsch, 2022), but researchers disagree as to whether this approach works better than appeals to personal benefits (Ashworth et al., 2021). It should be noted, however, that the present findings strongly suggest that when evaluating a public health message or other intervention during the current pandemic, one needs to consider its effects on both mitigation and vaccination, with the importance of the results for mitigation behaviors depending on the likelihood of breakthrough infections.

The present findings also illustrate one reason why researchers may observe disparate results in comparisons of prosocial and personal benefits, regardless of whether mitigation and vaccination are examined simultaneously or separately. As highlighted by the second component of the differential distress conundrum, younger and older adults respond differently with respect to psychological distress; not only has the COVID-19 pandemic produced different levels of distress in people of different ages, but depending on one’s age, the same level of distress may be associated with different decisions regarding mitigation and vaccination. Such results represent an issue known first in marketing and now in public health as market segmentation, and it is an obvious impediment to any one-size-fits-all intervention strategy.

Although it manifests itself here as differences between younger and older adults, this is likely just the tip of the iceberg with respect to market segmentation in public health during the COVID-19 pandemic. Further research will likely reveal other examples that need to be addressed to increase the effectiveness of interventions; but fortunately, the public health field has experience dealing with such problems (Evans, 2016). It has even been suggested that segmentation is not only just a problem to be solved, but also a tool to be used to promote healthy behavior during the pandemic (Meng and Olsen, 2021). As Evans et al. (2019) pointed out, the growth in social media and online communication has led to digital segmentation, which can be exploited for public health purposes just as it has been exploited in commercial marketing. While this approach has the potential to help in current efforts to motivate healthy behavior, at the same time it may also increase our understanding of the diversity and intersectionality of health behavior and people’s ability to cope with crisis.

## Data availability statement

The datasets presented in this study can be found in online repositories. The names of the repository/repositories and accession number(s) can be found at: osf.io/jmykd/.

## Ethics statement

The studies involving human participants were reviewed and approved by Institutional Review Board of Washington University in St. Louis. Written informed consent for participation was not required for this study in accordance with the national legislation and the institutional requirements.

## Author contributions

JM, MS, LG, and SH contributed to conception and design of the study. JM and MS performed the statistical analyses. JM wrote the first draft. All authors contributed to the article revision, read, and approved the submitted version.

## Funding

The research was supported by the National Institute on Aging of the National Institutes of Health under award number RO1AG058885.

## Conflict of interest

The authors declare that the research was conducted in the absence of any commercial or financial relationships that could be construed as a potential conflict of interest.

## Publisher’s note

All claims expressed in this article are solely those of the authors and do not necessarily represent those of their affiliated organizations, or those of the publisher, the editors and the reviewers. Any product that may be evaluated in this article, or claim that may be made by its manufacturer, is not guaranteed or endorsed by the publisher.
